# Freehand Ultrasound-Guided Transperineal Microwave Ablation for Benign Prostatic Hyperplasia: A Novel Application

**DOI:** 10.7759/cureus.96337

**Published:** 2025-11-07

**Authors:** Pedro Ivo C Ravizzini, Antonio João T de Aquino, Victor Arthur G Valdo, Daniel A Rangel, Roni C Fernandes

**Affiliations:** 1 Department of Interventional Radiology, Faculty of Medical Sciences of Santa Casa de Misericordia de São Paulo, São Paulo, BRA; 2 Department of Urology, Faculty of Medical Sciences of Santa Casa de Misericordia de São Paulo, São Paulo, BRA

**Keywords:** benign prostatic hyperplasia, ejaculation sparing, microwave ablation, mist, transperineal freehand ultrasound

## Abstract

The development of minimally invasive surgical therapies (MISTs) for benign prostatic hyperplasia (BPH) focuses on achieving effective lower urinary tract symptoms (LUTS) relief while preserving sexual function. While early investigations explored transperineal microwave application, we present the inaugural experience with a contemporary freehand ultrasound-guided transperineal microwave ablation (TPMA) technique as a novel MIST application. Two patients with symptomatic BPH underwent TPMA. Case 1 was a 54-year-old male (prostate volume 53 cc, International Prostate Symptom Score (IPSS) 27) seeking ejaculatory preservation. Case 2 was an 80-year-old male (prostate volume 69 cc) with a 15-month history of indwelling urinary catheter dependency. Procedures were performed under light intravenous sedation using a 16-gauge microwave antenna. Ablations were delivered at 20-30 W for 30-60 seconds per application using a freehand "moving shot" technique, under biplanar transperineal ultrasound guidance, maintaining an 8mm safety margin from the urethra and bladder neck, with an apical-sparing strategy. Procedures were successfully performed, with same-day discharge and catheter removal on postoperative day five. At six weeks, both patients showed marked improvement: Case 1: IPSS 27 to 7, Qmax 16.8 to 26 mL/s, with antegrade ejaculation preserved. Case 2: achieved catheter-free voiding (Qmax 27 mL/s, IPSS 8). Both were off alpha-blockers. No Clavien-Dindo ≥II complications occurred. Freehand ultrasound-guided TPMA appears to be a safe and feasible novel outpatient procedure for the treatment of BPH. This technique demonstrates promising short-term efficacy in relieving obstruction and may offer the potential for ejaculatory preservation. TPMA may represent a valuable addition to the MISTs arsenal, warranting further investigation in larger prospective studies.

## Introduction

Benign prostatic hyperplasia (BPH) is a prevalent condition among aging males, often leading to bothersome lower urinary tract symptoms (LUTS) that significantly impact quality of life [1]. While transurethral resection of the prostate (TURP) remains the gold standard, it carries inherent risks of bleeding, urethral stricture, and, notably, retrograde ejaculation in up to 90% of patients [2]. This has driven the development of minimally invasive surgical therapies (MISTs) that aim to reduce prostate volume with fewer complications and better preservation of sexual function [3].

Among these novel techniques, transperineal thermal ablation under ultrasound guidance has emerged as a promising approach. Recent studies have established the feasibility of this approach using laser energy (transperineal laser ablation (TPLA)) [4] and radiofrequency ablation (RFA) [5]. The application of microwave energy for prostatic tissue ablation has a historical precedent; a phase I study by Bartoletti et al. (2008) demonstrated the safety and creation of well-defined coagulative lesions using a single, fixed-position, cooled microwave applicator under transrectal ultrasound guidance in patients scheduled for subsequent open prostatectomy [6].

We describe a modern adaptation of this concept: the first application of a freehand ultrasound-guided transperineal microwave ablation (TPMA) technique for the treatment of BPH. This approach utilizes a percutaneous antenna without a fixed template, allowing for a dynamic "moving shot" application to precisely sculpt the ablation zone within the transition zone. Microwave ablation (MWA) is a well-established thermoablative technology that generates a rapid, predictable zone of coagulative necrosis, widely used in oncology [7-10]. Its application for BPH treatment via this specific freehand, transperineal ultrasound-guided approach represents a novel technical evolution in the MIST landscape. This report aims to demonstrate the technical feasibility and preliminary safety of this contemporary TPMA technique in two patients with BPH, as a precursor to larger efficacy studies.

## Case presentation

Case 1

A 54-year-old male presented with severe LUTS refractory to medical therapy (doxazosin and finasteride). His International Prostate Symptom Score (IPSS) [11] was 27, quality of life (QoL) score was 5, and he reported reduced ejaculatory volume. His maximum flow rate (Qmax) was 8.8 mL/s with a post-void residual (PVR) volume of 63 mL. Preoperative multiparametric magnetic resonance imaging (MRI) revealed a 53 cc prostate with no median lobe and a Prostate Imaging Reporting and Data System 2 (PIRADS 2) score. The patient expressed a strong desire to preserve antegrade ejaculation.

Case 2

An 80-year-old male with multiple comorbidities (hypertension, controlled type 2 diabetes) presented with urinary retention, requiring an indwelling urinary catheter for 15 months. He was deemed a high-risk candidate for conventional surgery. His prostate volume was estimated at 69 cc via MRI (PIRADS 2). The primary goal of treatment was to achieve catheter-free voiding.

Procedure

**Figure 1:**
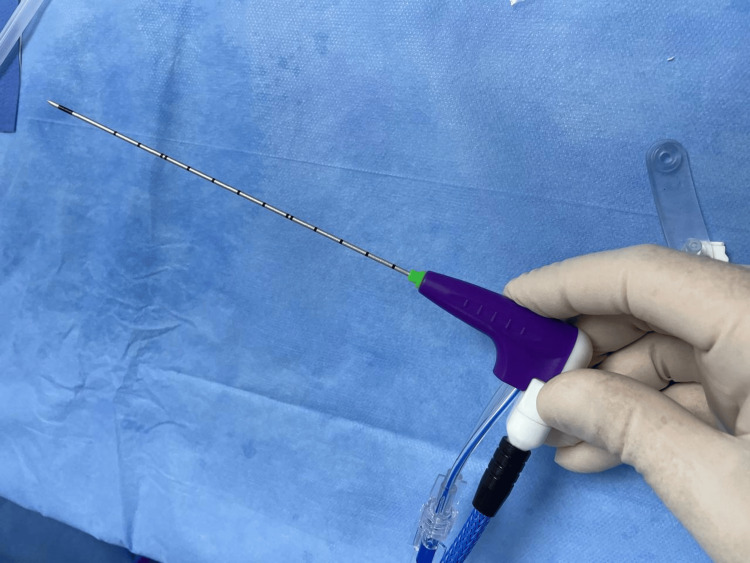
Microwave antenna, 16 gauge With the permission of ECO Medical Technology.

After institutional review board approval and obtaining written informed consent from both patients, the procedures were performed. The microwave ablation system (ECO Medical Technology, Nanjing, China) and 16-gauge antenna (Figure 1) were used in an off-label manner for this application, as they are currently cleared for general soft tissue ablation.

With the patients in the lithotomy position under light sedation (intravenous midazolam and fentanyl), prophylactic intravenous antibiotics (Ceftriaxone 2 g) were administered. An 18-gauge three-way urethral catheter was inserted for continuous saline irrigation (room temperature) to provide a thermal safety margin for the bladder neck.

A Mindray Resona i9 ultrasound system (Mindray, Shenzhen, China) with a biplanar transperineal probe was used for guidance. Using a freehand technique, a 15-cm 16-gauge microwave antenna (ECO Medical Technology) was percutaneously inserted into the prostate under real-time ultrasound guidance. The antenna was positioned within the hyperplastic transition zone. Ablation cycles of 30-60 seconds at 20 to 30 W power were delivered sequentially (Video 1). The power and time parameters were selected based on the manufacturer's pre-clinical characterization data for the antenna, which indicated that these settings would produce an approximately 1.0-1.5 cm elliptical ablation zone in soft tissue, allowing for overlapping ablations to treat the target volume while maintaining safety.

**Video 1:** Freehand transperineal ultrasound-guided microwave ablation procedure

Meticulous attention was paid to maintaining a minimum 8-mm safety margin from the prostatic urethra and bladder neck, a distance chosen based on known thermal spread profiles from the manufacturer's data and the continuous urethral irrigation. The transitional zone was preserved in the apical section of the prostate, up to the level of the verumontanum, to minimize the risk to the ejaculatory ducts (see Figure 1 for a schematic of the approach). A total of 11 and 12 ablation cycles were delivered for Case 1 and Case 2, respectively. Total procedure times (from first needle insertion to last needle withdrawal) were 25 and 30 minutes. A post-ablation study with SonoVue ultrasound contrast (Bracco, Milan, Italy) was performed to evaluate for satisfactory non-perfused ablation zones.

Outcomes

No intraoperative or immediate postoperative complications occurred. Both patients reported minimal pain (score 1/10 on a Visual Analog Scale [12]) and were discharged on the same day with a course of oral Cefuroxime 500 mg twice daily for seven days, Ketoprofen 150 mg daily for five days (analgesia), Prednisone 20 mg daily for five days (to reduce prostatic edema), and Doxazosin 4 mg daily (to prevent postoperative retention). The urinary catheters were removed in the office on postoperative day five after confirming a successful voiding trial.

At the six-week follow-up, patients demonstrated significant improvement (Table 1). Case 1 had an IPSS score that improved from 27 to 7, and a Qmax that increased from 8.8 mL/s to 26 mL/s. Most importantly, antegrade ejaculation was fully preserved, confirmed by patient report. Case 2 was voiding spontaneously with a Qmax of 27 mL/s and reported an IPSS score of 8. PVR was not measured at this visit. Both patients discontinued Doxazosin at six weeks. At three months post-TPMA, both patients reported sustained satisfaction with their urinary flow. No adverse events such as dysuria, significant hematuria (>24 h), urinary tract infection, or perineal discomfort beyond the first 48 hours were reported. According to the Clavien-Dindo classification, no complications ≥ Grade II were observed.

**Table 1:** Patient characteristics, procedural details, and outcomes IPSS: International Prostate Symptom Score, N/A: not available.

Parameter	Case 1	Case 2
Age (years)	54	80
Prostate volume (cc)	53	69
Indication	Medical therapy failure	Urinary retention
Pre-op IPSS/Qmax (mL/s)	27/8.8	N/A/indwelling catheter
Procedure time (min)	25	30
Ablation parameters		
Number of ablations	11	12
Post-op catheter duration	5 days	5 days
6-week IPSS/Qmax (mL/s)	7/26	8/27
Ejaculatory function	Preserved (Antegrade)	Not sexually active
Adverse events (Clavien-Dindo)	None	None

## Discussion

To our knowledge, this is the first report describing the use of a freehand ultrasound-guided TPMA technique with a dynamic "moving shot" approach for the treatment of BPH. Our initial experience suggests that this contemporary TPMA technique is a safe and technically feasible procedure with promising short-term functional outcomes. We explicitly frame this work as a proof-of-concept demonstration to guide future technical and clinical investigations.

The rationale for exploring MWA in this context is grounded in its distinct biophysical advantages, as previously explored in early feasibility studies [6]. However, our technique represents a significant methodological evolution. Unlike the single, fixed-position applicator used in the pioneering work by Bartoletti et al. [6], which created a single ~17 mm lesion per lobe, our freehand approach with multiple, sequential applications (20-30 W for 30-60 s) allows for customized and confluent ablation of the transition zone, potentially offering a more tailored treatment. The 20-30 W power and 30-60 s duration used here were selected to create controlled ablation zones suitable for this precise, overlapping application technique, while the continuous urethral irrigation and the mandated 8 mm margin ensured bladder neck and urethral safety.

Our short-term outcomes align with the growing evidence for transperineal ablation. The significant improvement in obstructive symptoms and flow rates is consistent with results reported for TPLA [4] and transperineal RFA [5]. Freehand TPMA may provide faster ablation of larger volumes, potentially reducing procedure time. Unlike RFA and TPLA, MWA is not limited by tissue charring. Furthermore, compared to the early fixed-probe microwave thermoablation [6], this freehand technique offers superior maneuverability for treating irregularly shaped glands and selectively sparing the apex. The paramount achievement in our first case was the preservation of antegrade ejaculation, accomplished by this apex-sparing technique and precise energy control.

However, we acknowledge that this technique introduces operator dependency. Future standardization could involve a transperineal template grid or guide to improve reproducibility, define a learning curve, and incorporate intraoperative checkpoints like real-time thermal monitoring at the margins. This report has significant limitations inherent to its design as a case report: it includes only two patients with a short follow-up. Long-term data on durability, recurrence, and precise rates of sexual function preservation are needed. We did not utilize validated sexual function questionnaires (e.g., Male Sexual Health Questionnaire to assess Ejaculatory Dysfunction (MSHQ-EjD), International Index of Erectile Function (IIEF)) or report PVR systematically, which should be included in future studies. Furthermore, the optimal ablation parameters and thermal dose (e.g., Cumulative Equivalent Minutes at 43°C (CEM43)) for MWA in the prostate require further elucidation through dedicated studies. The off-label use of the device and the associated regulatory pathway must be considered for wider adoption.

## Conclusions

Freehand ultrasound-guided transperineal microwave ablation represents a novel and technically feasible technique within the MIST landscape. Our initial experience demonstrates that TPMA is a safe outpatient procedure that can effectively alleviate bladder outlet obstruction secondary to BPH in the short term, with the potential to preserve ejaculatory function. The use of microwave energy offers specific thermodynamic benefits for prostate tissue ablation. These encouraging preliminary results warrant further investigation in larger, prospective cohort studies with longer follow-up to definitively establish the efficacy, durability, and role of TPMA in the management of BPH.
